# The Short-Term Efficacy of Bikini Incision and Traditional Incision in Total Hip Replacement for Elderly Patients *via* the Direct Anterior Approach

**DOI:** 10.3389/fsurg.2022.850046

**Published:** 2022-06-15

**Authors:** Qingsong Zhang, Bo Liu, Binghao Zhao

**Affiliations:** Department of Osteoarthrosis, Renmin Hospital, Hubei University of Medicine, Shiyan, China

**Keywords:** bikini incision, traditional incision, direct anterior approach, total hip replacement, hip replacement

## Abstract

**Background:**

The study aimed to explore the short-term clinical efficacy of bikini incision and traditional incision in total hip replacement *via* the direct anterior approach.

**Methods:**

The study enrolled 94 patients who underwent total hip replacement using the direct anterior approach between March 2018 and April 2020. They were assigned to the study group and the control group with 47 patients in each group using the random number table method. They received traditional incision and bikini incision, respectively. The operative time, intraoperative estimated blood loss, postoperative pain, length of hospital stay, incision healing, postoperative Harris score, and occurrences of complications were compared between the two groups.

**Results:**

There was no statistically significant difference in operative time, incision length, and intraoperative estimated blood loss between the two groups (*P *> 0.05). The length of hospital stay was shorter in the study group than that of the control group, and the difference was statistically significant (*P *< 0.05). There was no statistically significant difference in pain severity between the two groups (*P *> 0.05). No incision infection occurred in either group. The study group had small scar areas and scar scores than the control group (*P *< 0.05). There was no statistically significant difference in Harris scores between the two groups at three and six months postoperatively (*P *> 0.05). The rate of lateral femoral cutaneous nerve injury was lower in the study group than that of the control group, and the difference was statistically significant (*P *< 0.05).

**Conclusion:**

Bikini incision in total hip replacement *via* the direct anterior approach can shorten the length of hospital stay, promote incision healing, lower the incidence of complications, improve the prognosis, and promote recovery of patients, and it is worthy of being promoted for wide clinical use.

## Background

Total hip replacement is often used for the treatment of degeneration/arthritis and necrosis of the femoral head. Compared with the traditional total hip replacement, minimally invasive total hip replacement can reduce incision length, decrease intraoperative estimated blood loss, lessen muscle and tendon tissue injury, reduce the severity of postoperative pain, facilitate rapid recovery, and shorten the length of hospital stay (1). The progress of postoperative rapid recovery in total hip replacement patients is closely related to the surgical approach. Currently, the common surgical approaches clinically include the anterolateral approach, the posterolateral approach, and the direct anterior approach (2–5). The anterolateral approach and the posterolateral approach have been widely used at hospitals of various levels. Because of the more lateral position of the surgical incision, the greater trochanter has to be bypassed posteriorly or anteriorly during lateromedial exposure to reach the joint capsule and the femoral head, and the incision requires to be 10–15 cm. The incision is longer and excessive muscle tissues have to be dissected, the intraoperative estimated blood loss is larger, the wound is greater, and the time for postoperative ambulation and rehabilitation training is prolonged.

The study by Den et al. (6) showed that compared to traditional incision, the delayed wound healing rate is lower and the scar is smaller in bikini incision, and patient satisfaction is greater with bikini incision, while there is no difference between traditional incision and bikini incision in the extent of injury of muscles around the incision and the excellent rate of prosthesis position. Currently, the main principle of bikini incision is that it is parallel to the inguen, but there is consensus in the selection of the proper position of the incision. In the study by Leunlg et al. (7), the medial half of bikini incision was made in the inguinal fold and then extended laterally for half of the length. In the study by Manrique et al. (8), bikini incision was made 1 cm inferior to the inguinal fold, and the vertical line passing the anterior superior iliac spine was the center of the incision. In the study by Zhang et al. (9), a 7–10 cm incision was made laterally from the central inguinal skin fold. Because of the differences in bikini incision location, the relative distance is different from the incision to the lateral femoral nerve and the perineum and the location of muscles around the incision (tensor fasciae latae, rectus femoris, and sartorius) is different, and consequently, nerve injury and incision infection, the severity of muscle tractional injuries and operative time are different. Therefore, the location of bikini incision should be selected to make sure that the incision is parallel to the inguinal skin fold, exposes the surgical field, facilitates maneuvering by surgeons, reduces postoperative incision scar growth, and lessens intraoperative lateral femoral cutaneous nerve injury. Therefore, the study explored the short-term clinical efficacy of bikini incision and traditional incision in total hip replacement *via* the direct anterior approach.

## Materials and Methods

### Clinical Data

The study enrolled 94 patients who underwent total hip replacement using the direct anterior approach between March 2018 and April 2020. They were assigned to the study group and the control group with 47 patients in each group using the random number table method. The study was approved by the ethics committee of our hospital, and all patients had signed the informed consent. The study protocol was in accordance with Helsinki Declaration, and the methods were carried out in accordance with the relevant guidelines and regulations.

### Inclusion and Exclusion Criteria

Inclusion criteria were patients with (1) unilateral total hip replacement by the direct anterior approach and (2) good compliance and sanity.

Exclusion criteria were (1) history of hip surgery; (2) infectious disease; (3) hemiarthroplasty; (3) incapability to live unassisted preoperatively; (4) severe underlying disease; and (5) severe osteoporosis.

### Methods

#### The Control Group

A traditional incision was made. The patient was placed in the supine position and received general anesthesia combined with a lumbar plexus block. An 8–10 cm incision was made in parallel to and 3 cm from the line from the anterior superior iliac spine of the affected side to the lateral border of the patella. The skin and subcutaneous adipose tissue were incised, the deep fascia was opened longitudinally, and the gap between the tensor fasciae latae and the sartorius and rectus femoris was dissected bluntly. The branches of the lateral femoral artery were dissociated and ligated, and adipose tissue on the surface of the joint capsule was excised, and the joint capsule was dissociated laterally and opened to the gap between the gluteus minimus and gluteus medius. The gluteus minimus and the gluteus medius were pulled laterally and longitudinally dissociated along the medial rectus femoris, and the medio-inferior joint capsule was exposed. The joint capsule was incised in an “oblique L” or “T” shape in the glenoid labrum and the base of the femoral neck, and the femoral head and neck were fully exposed and osteotomy was performed along the base of the femoral neck and the femoral head and neck were extracted. The medio-inferior and latero-superior joint capsule was relaxed, and caution was exercised to protect the integrity of the insertions of the muscles of the lateral rotator group. The acetabulum was milled with a knife rasp until blood oozed beneath the cartilage of the acetabulum. The acetabulum was anteverted 12°, and the lateral acetabular inclination angle was 42°. The appropriately sized acetabulum and lining were assembled. The end of the operating table was lowered 45° to allow extension, adduction, and external rotation of the affected limb. The proximal femur was elevated using a greater trochanter hook and the osteotomy plane was exposed and a canal for the femoral stem was prepared using rasp handles of increasing sizes, and the femoral stem prosthesis and the femoral head were assumed and reduced. The deep fascia, subcutaneous tissues, and the skin were sutured layer by layer.

#### Study Group

The bikini incision was used in accordance with the standard practice provided by Manrique et al. (7), and the traditional incision was provided. The patient was placed in the supine position and received general anesthesia combined with a lumbar plexus block. An 8–10 cm oblique bikini incision was made 2 cm inferior to the inguinal skin fold and parallel to the line from the anterior superior iliac spine of the affected side to the lateral border of the patella. The skin and subcutaneous adipose tissue were incised, and the subcutaneous adipose tissues’ deep fascia were dissected bluntly. The deep fascia was opened along and in parallel to the line from the anterior superior iliac spine of the affected side to the lateral border of the patella and the joint capsule was exposed. Osteotomy was performed and the prosthesis was assembled, and the deep fascia was sutured as described in the control group. The incision was closed, and the subcutaneous adipose tissues and skin were sutured in parallel to the dermatoglyphics.

#### Postoperative Management

All patients were given a pneumatic pump for the affected limb postoperatively and received anticoagulation therapy for the prevention of lower limb vein thrombosis and analgesics for pain control. The patient started assisted ambulation guided by nursing staff on postoperative day (POD) 2 and was also told to start active flexion, extension, abduction, and adduction of the affected hip without limitation of the range of motion. To prevent infection and control pain, cefazolin and non-steroidal anti-inflammatory drugs were routinely injected at 1.0 g twice per day and twice daily. The discharge criteria of patients included adequate pain control on oral pain medication; independent transfer; ambulation of at least 200 ft alone; and the ability to climb stairs.

### Study Parameters

The operative time, intraoperative estimated blood loss, postoperative pain, length of hospital stay, incision healing, postoperative Harris scores, and occurrences of complications were recorded and compared by investigators who were blinded to group assignment and did not participate in the surgery.

The blood loss was calculated by subtracting the amount of normal saline from the amount of fluid in the aspirator, combined with the visual estimation method.

Incision healing included incision infection rate, scar area, and scar scores. Scar scoring (10) includes the color, thickness, vascular distribution, and softness of the scar. The total score is 15 points. The higher the score is, the more serious the scar will be.

Postoperative pain: The severity of pain was assessed using the visual analog scale (11) at POD 1 and 3 and three months postoperatively, with a score of 0–10. Higher scores indicated greater severity of pain.

Harris scores: The hip function was evaluated using Harris hip scores at three and six months postoperatively (9), which assessed the range of motion of the joint, deformity, function, and pain. The total score ranged from 0 to 100, and higher scores indicated better hip function, excellent ≥90, good 80–89, satisfactory 70–79, and poor <70.

Occurrences of complications: Complications including intraoperative lateral femoral cutaneous nerve injury, prosthesis dislocation, postoperative bone fracture around the prosthesis, brain embolism, and pulmonary embolism were recorded. As for the diagnosis of intraoperative lateral femoral cutaneous nerve injury, a nerve conduction examination was performed. At the same time, the timing of diagnosis of lateral femoral cutaneous nerve injury is very difficult. Most patients show unusual sensations in their thighs within a few months of surgery. The time point for diagnosis of lateral femoral cutaneous nerve injury in this study is unclear.

### Statistical Analysis

Data were analyzed using SPSS 21.0 software. The normality of data was analyzed, and the quantitative data were expressed in $\overset{®}{x}\pm s$ including operative time, intraoperative estimated blood loss, postoperative consumption of analgesics, length of hospital stay, and postoperative Harris scores and examined using the *t*-test. Categorical data were expressed as the rate (%) and examined using the chi-square (*χ*^2^) test. The sample size = 2[(U1 − *α* + U1 − *β*) S/*σ*]2, *α* = 0.05, *β* = 0.01, *n* = 91. According to the provisions of the State Food and Drug Administration, 15% is the shedding rate. *P *< 0.05 indicated a statistically significant difference.

## Results

### Comparison of the Baselines Between the Two Groups

The age of the study group ranged between 45 and 75 years with a mean age of 56.83 ± 10.23 years. There were 29 male patients and 18 female patients. Their body mass index (BMI) ranged between 22 and 28 kg/m^2^ with a mean BMI of 25.03 ± 3.23 kg/m^2^. The age of the control group ranged from 45 to 74 years with a mean age of 56.69 ± 10.16 years. There were 28 male patients and 19 female patients. The BMI ranged from 22 to 29 kg/m^2^ with a mean BMI of 25.12 ± 3.18 kg/m^2^. The two groups were comparable in the baseline variables (*P *> 0.05, Table 1).

**Table 1:** Comparison of general data between the two groups.

Variables	Study group (*n* = 47)	Control group (*n* = 47)	*t*/*χ*^2^	*P*
Male/female (*n*)	29/18	28/19	0.045	0.833
Age (years)	56.83 ± 10.23	56.69 ± 10.16	0.067	0.947
BMI (kg/m^2^)	25.03 ± 3.23	25.12 ± 3.18	−0.135	0.893
Site (*n*)				
Left	21	20	0.043	0.835
Right	26	27		
Diseases (*n*)				
Necrosis of the femoral head	17	16	0.190	0.909
Femoral neck fracture	15	14		
Congenital dysplasia of the hip	15	17		

### Comparison of Operative Time, Intraoperative Estimated Blood Loss, and Length of Hospital Stay Between the Two Groups

There was no statistically significant difference in operative time, incision length, and intraoperative estimated blood loss between the two groups (*P *> 0.05). The length of hospital stay was shorter in the study group than that of the control group, and the difference was statistically significant (*P *< 0.05) (Table 2).

**Table 2:** Comparison of operative time, intraoperative estimated blood loss, and length of hospital stay between the two groups $(\overset{®}{x}\pm s)$.

Groups	Operative time (min)	Incision length (cm)	Intraoperative estimated blood loss (mL)	Length of hospital stay (days)
The study group (*n* = 47)	83.29 ± 4.93	8.78 ± 1.02	168.29 ± 12.39	9.23 ± 2.01
The control group (*n* = 47)	81.98 ± 4.38	8.98 ± 1.01	165.93 ± 13.29	11.87 ± 2.31
*T*	1.362	−0.955	0.890	−5.911
*P*	1.176	0.342	0.376	<0.001

### Comparison of Pain Between the Two Groups

There was no statistically significant difference in pain severity between the two groups at POD 1, 3, and 7, and six months postoperatively (*P *> 0.05) (Table 3).

**Table 3:** Comparison of pain between the two groups $(\overset{®}{x}\pm s)$.

Groups	Postoperative day 1	Postoperative day 3	Postoperative day 7	Six months postoperatively
The study group (*n* = 47)	3.49 ± 1.29	2.57 ± 0.89	2.21 ± 0.81	0.67 ± 0.12
The control group (*n* = 47)	3.89 ± 1.37	2.89 ± 0.87	2.31 ± 0.78	0.69 ± 0.09
*T*	−1.457	−1.763	−0.610	−0.914
*P*	0.148	0.081	0.543	0.363

### Comparison of Incision Healing Between the Two Groups

No incision infection occurred in either group. The study group had small scar areas and scar scores than the control group, and the difference was statistically significant (*P *< 0.05) (Table 4).

**Table 4:** Comparison of incision healing between the two groups $(\overset{®}{x}\pm s)$.

Groups	Scar area (cm^2^)	Scar scores
The study group (*n *= 47)	1.02 ± 0.03	8.21 ± 0.39
The control group (*n* = 47)	9.21 ± 1.28	2.98 ± 0.23
*T*	−43.853	79.191
*P*	<0.001	<0.001

### Comparison of Harris Scores Between the Two Groups

There was no statistically significant difference in Harris scores between the two groups at three and six months postoperatively (*P *> 0.05) (Table 5).

**Table 5:** Comparison of Harris scores between the two groups $(\overset{®}{x}\pm s)$.

Groups	Three months postoperatively	Six months postoperatively
The study group (*n* = 47)	78.93 ± 5.01	86.03 ± 5.39
The control group (*n* = 47)	77.01 ± 5.12	84.39 ± 5.27
*T*	1.838	1.491
*P*	0.069	0.139

### Comparison of the Incidence of Complications Between the Two Groups

No prosthesis dislocation, postoperative bone fracture around the prosthesis, brain embolism, and pulmonary embolism occurred in either group. The rate of lateral femoral cutaneous nerve injury was lower in the study group than that of the control group, and the difference was statistically significant (*P *< 0.05) (Table 6).

**Table 6:** Comparison of the incidence of complications between the two groups, *n* (%).

Groups	Lateral femoral cutaneous nerve injury	Prosthesis dislocation	Postoperative bone fracture around the prosthesis	Brain embolism	Pulmonary embolism
The study group (*n* = 47)	5 (10.64)	0	0	0	0
The control group (*n* = 47)	15 (31.91)	0	0	0	0
*T*	6.351				
*P*	0.012				

## Discussion

With advances over the years, total hip replacement has become a very mature artificial joint replacement technique. As modern surgery has shifted to enhanced recovery, minimally invasiveness, and no specific postoperative complications, total hip replacement has also become increasingly refined, and anterior exploration of total hip replacement often uses the direct anterior approach and the posterior approach. Minimally invasive approaches have become a focus of clinical research to lessen the disturbance of muscles and soft tissues by surgery, reduce the occurrences of related complications, and promote postoperative rehabilitation, and comparative studies on different surgical approaches have been conducted but with conflicting results (7, 8). Therefore, the current study aimed to explore the short-term clinical efficacy of bikini incision and traditional incision in total hip replacement *via* the direct anterior approach.

The results of the current study showed that the operative time, incision length, and intraoperative estimated blood loss were comparable between the two groups (*P *> 0.05). The length of hospital stay was slightly lower in the study group than that in the control group (*P *< 0.05), suggesting that there was no difference between bikini incision and traditional incision in total hip replacement *via* the direct anterior approach in operative time, incision length, and intraoperative estimated blood loss, while the length of hospital stay was shorter with bikini incision, facilitated rehabilitation, and improved prognosis of the patients. The study by Zhang et al. (8) showed that compared to small anterolateral incision in initial total hip replacement, there was no noticeable difference from bikini incision in operative time, intraoperative estimated blood loss, postoperative drainage volume, postoperative transfusion rate, incision length, and length of hospital stay, which is consistent with our findings.

Total hip replacement can fully trim the acetabulum and acetabulum rim, and it should be done in parallel to the prosthesis border to avoid excessively concentrated postoperative stress, thus lessening postoperative local pain. The study by Cao et al. (12) showed that minimally invasive total hip replacement using the anterolateral approach can markedly lessen the severity of postoperative pain and promote fracture healing. The study by Schnatz et al. (12) showed that the severity of pain was comparable to bikini incision and traditional incision. Our results demonstrated that the severity of postoperative pain at POD 1, 3, and 7 and six months postoperatively was similar between the two groups (*P *> 0.05), which is consistent with previous findings, suggesting that the severity of pain is comparable in bikini incision and traditional incision in total hip replacement *via* the direct anterior approach. This is probably because the bikini incision is smaller in length, thereby reducing the severity of postoperative pain. The study by Leunig et al. (13) found that compared to traditional incision, bikini incision had smaller scar areas and lower scar scores, and patients had better appearances. The study by Lanting et al. (14) also demonstrated that bikini could enhance incision healing. Our results revealed that no incision infection occurred in either group, and the study group had smaller scar areas and lower scar scores than the control group (*P *< 0.05), suggesting better bikini incision healing in total hip replacement *via* the direct anterior approach. This is probably because bikini incision follows dermatoglyphics and effectively reduces scar formation. In addition, bikini incision is near the inguen and can be covered by underwear, and the postoperative scar is not easily seen, especially in female patients. It is esthetically more appealing and enhances patient satisfaction.

The postoperative recovery of hip function and the stability of structures around the hip are strongly associated with soft tissue injury, including muscles around the hip (sartorius, quadratus femoris, gluteus minimus, gluteus medius, rectus femoris, sartorius, and tensor fasciae latae) and the posterior joint capsule. The results of this study demonstrated that the two groups had comparable Harris scores at three and six months postoperatively (*P *> 0.05), suggesting that Harris scores were similar for bikini incision and traditional incision healing in total hip replacement *via* the direct anterior approach. This is probably because both incisions well preserve the muscles around the hip and the stable structures in the posterior joint capsule; in addition, bikini incision was mastered after traditional incision in total hip replacement *via* the direct anterior approach, and the surgeons have gained mastery of the anatomic structures in total hip replacement *via* the direct anterior approach and have accumulated rich experiences. Bikini incision and traditional incision only differ in the incision of the skin and the subcutaneous tissues while incision of the deep fascia and entry into the gap to expose the hip remain identical and soft tissue injuries are similar, which may be the main reason for the lack of difference in Harris scores between the two groups, which is similar to the findings by Lanting et al.

The study by Leunig et al. (13) showed that approximately 20% patients complained of hypoesthesia or tenderness of innervated areas of the lateral femoral nerve, and approximately 60% patients had hypoesthesia. The study by Bhargava et al. (14) is consistent with the above results. The results of this study demonstrated that no prosthesis dislocation occurred in either group, and the incidence of postoperative bone fracture around the prosthesis, brain embolism, pulmonary embolism, and lateral femoral cutaneous nerve injury was lower in the study group than that of the control group (*P *< 0.05), which are different from previous studies. The specific causes were as follows: (1) the lateral femoral cutaneous nerve formed from approximately 90% of the posterior rootlets of the L2 and L3 roots is less than 2 cm from the anterior superior iliac spine and travels downward below and at an angle of 83.38° with the inguinal ligament and enters the subcutaneous tissues at 3 cm from the inguinal ligament and distributes in the lateral femoral cutaneous region and continues its downward journal by sending out mediolateral branches, which could reach above the tensor fasciae latae (15). In the study by Leunlg et al. (6), the medial one-half of bikini incision was made in the inguinal fold and then extended laterally for one-half of the length. In the study by Manrique et al. (7), bikini incision was made 1 cm inferior to the inguinal fold, and the vertical line passing the anterior superior iliac spine was the center of the incision. In the study by Zhang et al. (8), a 7–10 cm incision was made laterally from the central inguinal skin fold. In this study, bikini incision was made approximately 4 cm inferior to the anterior superior iliac spine or 2 cm inferior to the inguinal skin fold and just lies above the skin where the lateral femoral cutaneous nerve enters the subcutaneous tissues, which avoids direct injury to the lateral femoral cutaneous nerve when the subcutaneous tissues are incised. The deep fascia is exposed laterally and longitudinally by bluntly dissecting the subcutaneous tissues, and the deep fascia was then incised for about 10 cm at 3 cm lateral to and parallel to the line connecting the anterior superior iliac spine and the lateral border of the patella and the gap between tensor fasciae latae and sartorius and rectus femoris was bluntly dissected and exposed and caution should be exercised to avoid injury to the medial-lateral femoral cutaneous nerve and its distal lateral branches. (2) when the fascia is sutured, the needle is entered at a distance of 1–2 cm from the margin and caution should be taken to avoid injury to the lateral femoral cutaneous nerve and avoid suturing and ligation of the nerve leading to entrapment syndrome, which manifests as hypoesthesia or pain in the anteromedial thigh (16). A direct approach may cause reversible lateral femoral cutaneous nerve injury but symptoms will gradually disappear over time, which is closely associated with excessive traction of the lateral femoral cutaneous nerve for better exposure of the acetabulum and femoral stem assembly and is partially due to direct nerve transection; for these patients, symptoms persist, indicating that intraoperative excessive traction of adjacent soft tissues should be avoided, thus reducing the rate of lateral femoral cutaneous nerve injury.

### Limitations

Because of the small sample size and short study duration, and postoperative recovery was not completely examined, further studies in the multi-centers are required for confirmation of the study findings. In addition, there were many slight differences among the bikini incision, we only tested one method in the study. More incision methods should be conducted and compared.

## Conclusion

In conclusion, the operative time, intraoperative estimated blood loss, pain severity, and Harris scores are comparable in bikini incision and traditional incision in total hip replacement *via* the direct anterior approach; however, bikini incision has advantages in the length of hospital stay, incision healing, and occurrences of complications and is worthy of wide clinical application.

## Data Availability

The raw data supporting the conclusions of this article will be made available by the authors, without undue reservation.
